# A qualitative study on the impact of hemophilia on sexual functioning and well-being in Dutch men

**DOI:** 10.1186/s41687-025-00946-6

**Published:** 2025-09-29

**Authors:** Tessa C. M. van Gastel, Lorynn Teela, Vicky Lehmann, Martijn R. Brands, Jan P. Marchal, Kees Gravendeel, Marjolein Peters, Karin Fijnvandraat, Lotte Haverman

**Affiliations:** 1 Amsterdam UMC Location University of Amsterdam, Emma Children’s Hospital, Child and Adolescent Psychiatry & Psychosocial Care, Amsterdam, The Netherlands; 2 Amsterdam Public Health, Mental Health and Health Behaviors & Chronic Diseases, Amsterdam, The Netherlands; 3 Amsterdam Reproduction and Development, Child Development, Amsterdam, The Netherlands; 4 Amsterdam Public Health, Mental Health and Digital Health, Amsterdam, The Netherlands; 5 Amsterdam UMC Location University of Amsterdam, Department of Medical Psychology, Amsterdam, The Netherlands; 6 Amsterdam UMC Location University of Amsterdam, Emma Children’s Hospital, Paediatric Hematology, Amsterdam, The Netherlands; 7 Dutch Hemophilia Patient Association, Nijkerk, The Netherlands

**Keywords:** Men, Hemophilia, Sexual functioning, Chronic disease, Qualitative study, Lived experiences, HIV

## Abstract

**Background:**

Previous research shows that hemophilia negatively affects sexual functioning and well-being in men with hemophilia. To gain deeper understanding of the impact of hemophilia on sexual functioning and well-being, we conducted this qualitative study.

**Methodology:**

Experiences and needs of men with hemophilia were gathered through focus groups and semi-structured interviews and then thematically analyzed using MAXQDA.

**Results:**

Fourteen men with hemophilia (median age 53 years, range 22–71) participated. They reported both hemophilia-specific factors (e.g., bleeding or joint limitations) and chronic disease factors (e.g., pain or self-esteem) negatively affecting sexual functioning and well-being, with differences in the factors and the degree of impact observed based on the age of the men with hemophilia. For instance, older men (aged over 40 years) reported more mental and physical complaints compared to younger men (aged 40 or younger). Many men were uncertain about how to address sexual functioning.

**Conclusions:**

Tools (e.g., patient reported outcome measures, information materials, training) to provide and assess information about sexual functioning and well-being, to normalize the topic and to open up discussions between men with hemophilia and healthcare professionals are necessary. Future research should focus on developing tools for this purpose.

## Background

Hemophilia is an X-linked hereditary bleeding disorder primarily affecting males, characterized by a deficiency in clotting factor VIII (hemophilia A) or factor IX (hemophilia B), and resulting in an increased tendency to bleed [1]. Three severity levels of hemophilia are distinguished based on clotting factor deficiency: severe, moderate, and mild. Severe hemophilia may cause easy bruising, pain, excessive bleeding following trauma or surgery, and loss of joint movement due to recurrent bleeds in joints and muscles [2, 3]. The treatment of hemophilia has greatly improved over the past five decades. In the 1970s, concentrates containing factor VIII or IX became available in high-income countries. This was a significant advancement in hemophilia care, as prior treatment consisted of blood transfusions and the use of fresh frozen plasma at the hospital. This advancement enabled home-based hemophilia treatment, as the concentrates could be stored at home. Unfortunately, in the years thereafter men with hemophilia (MWH) faced an increased risk of human immunodeficiency virus (HIV) (until 1985) and hepatitis C virus (HCV) (until 1990) infections due to contaminated clotting factor products [1, 4, 5]. Current viral inactivation techniques and recombinant products have eliminated the risk of contracting HIV and HCV through blood products [4–6]. For those with severe hemophilia, treatment consists of prophylaxis with factor concentrates or non-replacement therapy. People with moderate of mild hemophilia receive on-demand treatment in case of bleeding [2, 3]. Novel treatments, including gene therapy, are becoming increasingly available in high-income countries [5, 7, 8].

Advancements in treatment and availability of novel treatment options have significantly improved medical outcomes for patients with hemophilia. These developments have also increased awareness of the broader impact of hemophilia on daily life, health-related quality of life (HRQOL), and the importance of comprehensive care for patients with hemophilia [3, 6, 9–12]. Studies in the Netherlands report that the advancements in treatment led to decreased bleeding rates and improved joint health especially in young MWH. However, many patients with severe hemophilia continue to experience joint bleeds, joint deterioration [6, 13], and challenges in daily life due to joint pain [6].

Sexual functioning (e.g., sexual arousal, orgasm) and sexual well-being (e.g., positive feelings about sexual activity), together constitute an important aspect of daily life, and is also negatively affected by hemophilia [14–16]. Previous research reports that due to hemophilia-related pain, MWH experience a lower ability and desire to have sex in comparison to people without hemophilia [14]. Additionally, bleeds may occur as a result of sex in particular positions [16, 17], and limited joint movement may restrict positions during sexual activity [14, 16, 18]. Despite these findings, prior studies on men’s sexual functioning have focused on physical aspects, excluding sexual well-being. Many of these studies relied primarily on quantitative binary assessments of physical restrictions [18] or focused on the reports or perspectives of healthcare professionals (HCPs) rather than patients [14, 19]. While some qualitative studies have explored the lived experiences of MWH, they rarely focused on sexual functioning and well-being exclusively. Instead, sexual functioning and well-being emerged as minor themes within broader discussions, such as living with HIV [20] or challenges related to disclosing a hemophilia diagnosis in intimate relationships [21].

Previous studies report that provision of information and communication between a patient and HCP about sexual functioning and well-being is crucial but scarce [16, 18, 22, 23]. Both patients and HCPs find it challenging to address this topic. Barriers to these discussions include the assumption that there is no time to discuss the topic at a routine medical appointment, the fear no (medical) treatments can be offered regarding hemophilia-related problems in sexual functioning and well-being [14] or concerns about feelings of discomfort when discussing the topic [14, 17, 22]. However, discussing restricted sexual functioning and well-being can maximize care benefits [14], as it can help to identify problems and allow patients and HCPs to find solutions to improve care outcomes.

We conducted this qualitative study to gain detailed insights into men’s experiences on how hemophilia influences their sexual functioning and well-being, and to identify their communication needs regarding this topic.

## Methods

### Participants and procedure

Eligible participants were adult (i.e., older than 18 years) Dutch-speaking MWH with all hemophilia severities. Patients who are registered at the Hemophilia Treatment Center (HTC) of the Amsterdam University Medical Centers (Amsterdam UMC) were mailed an invitation letter, and the Dutch Hemophilia Patient Association (NVHP) posted an invitation on its social media platforms inviting men to participate in focus groups or semi-structured interviews. Considering the availability of viral inactivation techniques, which mostly eliminated the risk of HIV (1985) and HCV (1990) infections via hemophilia treatment, the focus groups were split into men over 40 years and men aged 40 or younger. Throughout this manuscript, men over 40 years are referred to as older MWH and men aged 40 or younger as younger MWH. A semi-structured interview was offered as a choice for participants who might feel uncomfortable discussing sexual functioning in a group setting. Written informed consent was provided by all participants before participation. Participants received a gift card of €10. Data collection took place from June to November 2023, continuing until the data was sufficient, with overlapping insights among participants, providing enough depth. Approval of the Medical Ethical Committee of the Amsterdam UMC was obtained (W22_164 # 22.213).

### Characteristics of participants

Sociodemographic (e.g., relationship status, age, education) and clinical data (e.g., severity of hemophilia, clotting factor percentage, comorbidities) were collected through a self-created questionnaire and from medical records (clinical data only and if consented). The questionnaire also asked about restrictions in daily life due to hemophilia: ‘Do you feel restricted in daily life due to your bleeding tendency?‘. The response options were ‘yes,’ ‘sometimes,’ and ‘no.’ Additionally, an open-ended question was included to gather more details on how MWH feel restricted. Sexual orientation, culture and religion were not collected as part of the patient characteristics in this study.

### Data collection

The focus groups or semi-structured interviews were conducted in person at the Amsterdam UMC or online, based on the participants’ preferences. Each focus group was led by two experienced clinicians (JPM, psychologist; MB, medical doctor/researcher) who alternated between being interviewer or observer. Interviews were conducted one-on-one by one of the clinicians (JPM or MB). Both clinicians are experienced in qualitative interview techniques and familiar with the population. They were both male and had no treatment relationships with participants to create a more comfortable situation. The focus groups took around 120 min and the semi-structured interviews took around 60 min.

To create a safe and non-judgmental environment, focus groups and interviews started with underlining the importance of keeping personal information confidential. A conversation guide was developed to focus on five themes regarding sexual functioning and well-being: (1) relationship/dating, (2) physical complaints, (3) medication, (4) reproductive choices, and (5) communication with HCPs. These themes were based on literature [14, 16–19, 22] and expert experience. The conversation guide provided structured prompts and guidance throughout the focus groups and interviews to help the interviewers maintain a comfortable and open dialogue. Additionally, open-ended follow-up questions were included to ensure important aspects were addressed, while allowing flexibility to adapt to the flow of the discussion and keep the focus on the five themes. In all focus groups, the participating men were asked to write down their thoughts about these five themes on post-its or a digital equivalent, which were organized by content and subsequently discussed in the group. This is widely considered an effective way to enhance engagement, elicit opinions from all participants about a sensitive topic, and improve understanding of different perceptions [24]. Post-its were not used during the semi-structured interviews, only the open-ended questions from the interview guide were used. To ensure the data met the study’s aim, the content of the semi-structured interviews and focus groups was periodically evaluated with the interviewers.

### Analyses

The Statistical Package for Social Sciences (SPSS) version 28.0 was used to perform descriptive analyses to characterize all participants. The focus groups and semi-structured interviews were audio recorded, transcribed verbatim and analyzed in MAXQDA Plus 2022 [25] using thematic analysis. This occurred in the following steps: (I) familiarizing with the data and highlighting relevant text fragments, (II) grouping relevant text fragments to generate initial codes, (III) sorting the different codes into the pre-defined themes of the conversation guide (deductive coding) and identifying subthemes (inductive coding), (IV) reviewing the (sub)themes and (V) defining the final (sub)themes [26]. If necessary, the pre-defined themes were adjusted. Two researchers (TvG and NvS) conducted the analysis, with one researcher (TvG) coding all transcripts and 22% being double coded by (NvS) independently. TvG and NvS compared and discussed their coding to resolve disagreements. Remaining disagreements were discussed with a third researcher (LT) to reach consensus. Finally, a patient advocate (KG, male, 69 years, severe hemophilia A, not participating in focus group or semi-structured interview) from the NVHP was asked to provide feedback regarding the interpretation and completeness of the results. This strengthens the results and ensures that the findings accurately reflect patients’ experiences [27], particularly of the MWH community. The subthemes are reported in an order that reflects their discussion frequency.

## Results

### Participant characteristics

In total, 14 MWH participated in four focus groups (three groups consisted of two men each, and one group consisted of three) and five semi-structured interviews. The median age was 53 years (range 22–71). A total of 50% had mild, 21% moderate, and 29% severe hemophilia. Most participating men (57%) reported that they did not feel restricted and almost half (43%) of the men did (sometimes) feel restricted in daily life because of their bleeding tendency, due to physical impairments (e.g., limitations in movement, difficulty walking) and having to be alert and careful all the time. Participant characteristics are presented in Table 1.

**Table 1:** Participant characteristics (*n* = 14)

		Median	SD
Age in years (range 22–71 years)		53	15.6
Age group		n	%
	> 40 years	10	21
Bleeding disorder		n	%
	Hemophilia A	11	79
	Mild	5	36
	Moderate	3	21
	Severe	3	21
	Hemophilia B	3	21
	Mild	2	14
	Moderate	0	0
	Severe	1†	7
Past/current HIV/HCV infection§		n	%
	HIV positive	3	21
	HCV positive	4	29
Comorbidity‡		n	%
	Yes	5	36
	No	9	64
Recruitment through		n	%
	Amsterdam UMC	12	86
	Via NVHP	2	14
Educational level¶		n	%
	Secondary education	4	29
	Tertiary education	10	71
Relationship status		n	%
	Partnered	10	71
	Single	4	29
Do you feel restricted in daily life due to hemophilia?		n	%
	Yes	4	29
	Sometimes	2	14
	No	8	57

† Currently normal clotting factor levels, post-transplantation (liver)

‡ Chronic obstructive pulmonary disease (*n* = 1), arthritis (*n* = 1), pulmonary hypertension (*n* = 1), and unknown (*n* = 2)

§ Reported during focus group or semi-structured interview only

¶ Participants highest completed level of education. Education level was categorized according to ISCED levels: Primary education (ISCED level 1), Secondary education (ISCED levels 2 and 3), Tertiary education (ISCED levels 6 and 7)

### Focus groups and semi-structured interviews

The themes identified from the focus groups and semi-structured interviews are described below. The themes identified within each pre-defined theme are reported in an order that reflects their discussion frequency. Associated quotes per subtheme are presented in Table 2. These quotes are numbered and referenced throughout the results section.

**Table 2:** Overview of the identified themes and associated quotes

Relationship/dating life	
Disclosure about diagnosis	⋅ *(Q1) When I go hiking with a partner*,* it is important she is aware of my hemophilia for practical reasons.* 31 years, mild ⋅ *(Q2) It* [hemophilia] *is not something that dominates my life*,* therefore it is not something I disclose immediately in* [the early stages of] *a relationship.* 47 years, mild
Impact of hemophilia	⋅ *(Q3) When someone is with me very often*,* as is the case in a relationship*,* then this person will truly experience what the impact* [of hemophilia] *is on my life.* 22 years, severe ⋅ *(Q4) The life expectancy for individuals with hemophilia around the 1960s was a factor that made it difficult to get into a relationship.* 71 years, severe
Partner support	⋅ *(Q5) Sometimes she* [my partner] *gets nervous and takes it* [hemophilia] *into account a bit too much.* 41 years, severe ⋅ *(Q6) My partner has her own [medical] problems. It was a huge relief we both had ‘issues’.* 63 years, mild
HIV	⋅ *(Q7) The first thing I thought was; oh shit*,* I hope I didn’t infect my wife [with HIV].* 58 years, severe ⋅ *(Q8) I still have to deliver* [to a partner] *that heavy message about HIV and*,* to a much lesser extent*,* hemophilia.* 63 years, moderate
**Physical functioning**	
Bleeding	- *(Q9) We have fun together* [sexually] *and if that leads to unpleasant consequences* [bleedings], *then I’ll deal with that afterwards.* 66 years, severe - *(Q10) After that incident* [blood in urine], *I became a bit more cautious in my movements during sexual activity*,* but I quickly resumed acting normally.* 46 years, mild
Sex positions	- *(Q11) Engaging in sexual activity in the missionary position becomes challenging when your ankle joints have limited mobility.* 63 years, mild - *(Q12) You become more innovative*,* exploring alternative sex positions and incorporating aids or toys that you might not typically use.* 58 years, severe
Pain	- *(Q13) I noticed my desire to have sex decreased because of the chronic pain I had.* 41 years, severe - *(Q14) Experiencing pain during sexual activity can be distracting*,* making it less fun.* 63 years, moderate
**Mental functioning**	
Impact of mental functioning	- *(Q15) I lost my erection because I became too preoccupied with my thoughts.* 71 years, severe - *(Q16) I felt* [when in pain] *as though my body was inferior.* 63 years, moderate
**Medication**	
Impact of medication	- *(Q17) The option of home treatment provides freedom*,* not just in sexual context*,* but also in other aspects of daily life.* 66 years, severe - *(Q18) I won’t restrict myself to having sex only when I’ve had sufficient treatment.* 41 years, severe
**Reproductive choices**	
Reproductive choices	- *(Q19) With the current advancements in hemophilia treatment*,* living a normal life is entirely possible. Therefore*,* I don’t see any reason to refrain from having children due to hemophilia.* 33 years, mild - *(Q20) For me*,* hemophilia felt like a serious condition*,* something I wouldn’t want to pass on.* 63 years, mild
Discussing reproductive choices • With healthcare professional • With partner	- *(Q21) We were provided with information by our doctor*,* particularly after expressing our desire to have a child.* 38 years, moderate - *(Q22) Our conversation turned to wanting children. I mentioned my thoughts about hemophilia inheritance. She was struck by the idea of ‘imperfect’ daughters and ended our relationship.* 31 years, mild
**Communication with healthcare professional**	
Experiences discussions about sexual functioning and well-being	- *(Q23) During my appointment*,* we discussed various aspects related to hemophilia*,* but we didn’t specifically address how these aspects might affect sexual activity.* 66 years, severe - *(Q24) When I had the iliopsoas bleed*,* they [HCPs] informed me that it could be a result of being sexually active. They provided me with more information*,* but once my symptoms subsided*,* I didn’t receive any further information.* 38 years, moderate
Needs for discussions about sexual functioning and well-being	- *(Q25) I wouldn’t mind if the doctor brings up the topic* [sexual functioning], *as long as I can express that I prefer not to discuss it.* 22 years, severe - *(Q26) It might be beneficial to discuss the impact of limited physical function on sexual activity with a psychologist.* 63 years, mild
How to initiate and address sexual functioning and well-being	- *(Q27) When using a questionnaire*,* some might simply check ‘no’ to avoid discussing the topic further.* 33 years, mild - *(Q28) It shouldn’t be a recurring topic in every outpatient visit*,* but it could be addressed during specific phases of life.* 66 years, severe

Note: All quotes were translated from Dutch into English

#### Relationship/dating

When elaborating on relationships and dating, participating MWH described experiences that clustered into four themes: (1) disclosure about diagnosis, (2) impact of hemophilia, (3) partner support, and (4) HIV. Most men reported a heteronormative preference.

Some of the MWH reported *disclosing their diagnosis* immediately or at an early stage of a relationship. Reasons for disclosure included that it is important to be open in a relationship, that it is noticeable that they have a disability, or for practical reasons (e.g., knowing emergency procedures) related to their diagnosis (Q1). Most of the MWH reported that they do not feel ashamed about disclosing their diagnosis. Some participants stated they only disclose their diagnosis if they see a possible future with the person they are dating. Other men stated they did not choose to disclose their diagnosis, mainly if they do not feel affected by the diagnosis in daily life (Q2). They do disclose their diagnosis when the conversation moves to the topic naturally. Negative responses from dating partners were rare.

Most MWH were aware of the *impact of hemophilia* on their relationship and dating life, which becomes more apparent to partners as a relationship evolves and grows closer (Q3). However, the majority of MWH expressed a desire not to constantly focus on their diagnosis. Nevertheless, some level of caution is occasionally necessary (e.g., to prevent from pain or bleeds). A few older men mentioned having fewer relationships as a result of their hemophilia or feeling less confident about their chances in their dating life (Q4).

Regarding the *support of a partner*, MWH emphasized the importance of their partner understanding their diagnosis. Some MWH noted that their partner is overly cautious (Q5), for example due to lack of knowledge about impact of the diagnosis. Furthermore, some MWH reported their partners also had a medical condition, which contributed to understanding each other (Q6).

Consequences of *HIV* and/or HCV infections were only shared by older participants. Those living with HIV mentioned they felt anxious about potentially having transmitted the virus to their partners (Q7). Furthermore, dating lives were negatively affected by an HIV infection. MWH living with HIV were less actively dating, or relationships ended upon disclosing their HIV-positive status. Consequently, disclosing the HIV infection was more difficult than disclosing their hemophilia (Q8). Some men experienced impact on their sexual functioning and well-being due to anxious and insecure feelings, even 40 years after the HIV diagnosis.

#### Physical and mental health issues

Physical complaints that are a result of hemophilia can negatively affect sexual functioning. During the focus groups and semi-structured interviews, three themes were identified: (1) bleeding, (2) sex positions, and (3) pain. In addition to these physical aspects, discussions during the focus groups and semi-structured interviews revealed that (4) mental health issues can also influence sexual functioning. This was therefore included as an additional theme.

*Bleeding* sometimes occurred during or as a result of sexual activity, which included hematospermia (i.e., blood in sperm), bleeding of the glans of the penis, iliopsoas muscle bleed, and tongue bleed after kissing. Most men stated they would rather enjoy sexual activity than be cautious (Q9), while some others said they became extra cautious after a bleeding had happened (Q10). Most men did not feel ashamed when experiencing bleeding.

Regarding *sex positions*, mostly MWH older than 40 years reported that not all positions were possible due to limited joint movement (Q11). Despite these limitations, most participants still engaged in sexual activity and reported being more inventive in finding sex positions that work for them (Q12). Additionally, some men mentioned that an active lifestyle helps them remain physically capable of being sexually active.

*Pain* was another factor that negatively affected the variety of sex positions. Some MWH mentioned that experiencing pain decreases their desire to have sex at all (Q13) or that it is distracting during sexual activity (Q14). Furthermore, pain can have a negative impact during dates involving physical activity.

The impact of *mental aspects* on sexual functioning was primarily mentioned by older MWH. Feelings of anxiety, stress, and insecurity (e.g., concerns about being ‘a good enough partner’) can be a result of their hemophilia, which negatively affects sexual functioning and well-being. For example, some MWH mentioned that they are not able to sustain an erection due to stress (Q15), while others feel insecure due to their disease or constant experience of pain (Q16). Men aged 40 or younger did not mention such mental effects on their sex lives.

#### Medication

Improved treatment of hemophilia (e.g., prophylactic treatment to reduce the risk of bleeds) and availability of home-based treatment increased feelings of freedom and trust, also positively affecting their sex lives (Q17). None of the MWH reported scheduling sexual activity around their prophylactic treatment (Q18). A few older men reported side effects of medication for pain or HIV (e.g., fatigue, headache) negatively affecting their desire to be sexually active.

#### Reproductive choices

Regarding reproductive choices, two themes were identified from the focus groups and semi-structured interviews: (1) reproductive choices and (2) discussing reproductive choices (with a partner or HCP).

Most MWH stated hemophilia and the possibility of genetically transmitting this bleeding disorder did not affect their *reproductive choices*. Especially younger MWH did not have concerns about having children and passing on hemophilia as it did not impact their life due to improved treatment (Q19). A few older MWH disclosed a deliberate choice of not having children because of the risk of genetically transmitting hemophilia to potential female offspring (Q20).

Regarding *discussing reproductive choices*, most MWH reported they received clear information from their HCP (Q21). Additionally, discussing reproductive choices with (dating) partners was also reported. While these discussions often went well, a few men reported that information about the heredity of hemophilia led to a breakup or an uncaring response (Q22).

#### Communication with healthcare professionals

From the discussion about communication with HCPs about sexual functioning and well-being, three themes were identified: (1) experiences with discussions about sexual functioning and well-being, (2) needs for discussions about sexual functioning and (3) advice on how to effectively initiate conversations about sexual functioning and well-being and address the topic.

The overall experiences of MWH indicated that *discussions about sexual functioning and well-being do* not often take place with an HCP (Q23). If they occurred, discussions with HCPs are mostly medically oriented, and sexual functioning and well-being is only addressed when there is a specific medical reason (e.g., hematospermia, iliopsoas bleed) (Q24). Consequently, some men mentioned they searched for information themselves (e.g., online or with other patients). A few men expressed their beliefs about the lack of discussions, which included that HCPs either assume patients have no sexual complaints or that they already have all necessary information.

Opinions differed on whether *discussions about sexual functioning and well-being* were *needed*. While most MWH stated that is generally important to be able to discuss sexual functioning and well-being with an HCP, a substantial number also mentioned that such discussions were not necessary for them personally (Q25). Furthermore, while most men stated that the HCP should initiate and lower barriers regarding the discussion about sexual functioning and well-being, some mentioned that patients themselves should start the discussion about this topic. All MWH agreed that sexual functioning and well-being is a sensitive topic and that it is important to be aware of this sensitivity. Some men suggested having discussions about sexual functioning with someone other than their medical doctor, such as the hemophilia-nurse or a psychologist to address emotional implications of hemophilia on sexual functioning and other aspects of daily life (Q26).

Specific *advice on how to initiate and address sexual functioning and well-being* also varied. Some MWH mentioned a questionnaire to screen for sexual difficulties would be helpful, whereas other men stated that initiating the discussion face-to-face is preferred. They further specified that patients may tend to falsely indicate that everything is well on a questionnaire (Q27). Furthermore, providing informational material to take home was mentioned as helpful, and linking information to different life phases (e.g., puberty, moving out of parents’ home, when considering having a children) was considered valuable (Q28).

### Patient advocate perspective

The patient advocate recognized the different aspects that were discussed during the focus groups and semi-structured interviews. He emphasized the disparities in experience based on the age of the MWH and stressed the importance of being aware of these variations. Additionally, the patient advocate acknowledged the reluctance to discuss sexual functioning and well-being with HCPs and suggested a tool (e.g., a visual aid that serves as a focal point for conversation) to facilitate open discussion about sexual functioning and well-being.

## Discussion

This qualitative study is among the first to explore the impact of hemophilia on sexual functioning and well-being, as well as the communication needs of MWH. Overall, an impact of hemophilia on sexual functioning and well-being was reported. Experiences shared in the current study regarding the impact of hemophilia included the need to be cautious during sexual activity occasionally and that a partner is sometimes (too) cautious during sexual activity. MWH reported physical complaints that negatively influenced sexual functioning and well-being, as well as mental complaints, such as feelings of anxiety, stress and insecurity. Participants also mentioned the positive effects of improved hemophilia treatment on their sexual functioning and well-being.

Differences in experiences were observed between MWH over 40 years of age and younger MWH. Older MWH reported greater impact on sexual functioning and well-being due to physical and mental complaints, the burden of disclosing an HIV-infection, and challenges regarding reproductive choices than younger MWH. We expect this to be the result of the historical variations in treatment, resulting in more severe joint limitations, pain [1–4, 6–8, 28], and the chance of infection with HIV and HCV by contaminated clotting factor products in older MWH [1, 4, 6]. Advancements in treatment may have reduced the differences in symptoms between severities of hemophilia. Older MWH, who received less treatment, often experienced more severe symptoms. In contrast, younger MWH, benefiting from improved treatment, reported fewer symptoms or symptoms similar to mild hemophilia.

The factors influencing sexual functioning and well-being identified in the current study can be considered both hemophilia-specific (e.g., bleeds, and inheritance) and factors more generally applicable to individuals with chronic diseases in general (e.g., disclosure of diagnosis, an overly cautious partner, insecurity, and pain). Similar factors (e.g., insecurity and pain) have been reported in review studies on different chronic diseases, including rheumatic diseases (where arthritic symptoms may overlap with those seen in hemophilia) and neurological diseases [29, 30]. Given the diversity of these factors, it is important to have a broad focus on the impact of a chronic disease on sexual functioning and well-being. Verschuren and colleagues [31] developed a generic conceptual framework about the impact of a chronic disease on sexual functioning and well-being. This framework emphasizes that the impact of a chronic disease on sexual functioning and well-being extends beyond physical symptoms, with psychological and relational distress playing a crucial role [31]. Sexual functioning and well-being can occur together but can also occur independently. For instance, MWH in the current study described how limited joint mobility restricted certain sexual positions (sexual functioning), but also shared feeling inventive to maintain satisfying sexual activity (sexual well-being). Paying attention to both aspects of sexual functioning and sexual well-being is emphasized by this finding.

The impact of hemophilia on sexual functioning and well-being is not often discussed with HCPs, as was also found in a nationwide study in Dutch adult MWH [15]. Communication needs regarding discussing sexual functioning and well-being varied among participants. While MWH recognized the value of discussing these topics, most did not consider these discussions necessary for themselves. This intriguing finding, not further explored in the current study, may reflect embarrassment regarding discussing these sensitive topics or a lack of familiarity with such discussions. Additionally, most of these men did not yet experience restrictions in sexual functioning and well-being and might not recognize the preventive or informative value of such discussions. The participants mentioned that HCPs initiating the topic and taking away uneasiness or embarrassment can help MWH feeling more comfortable raising and discussing the topic. This should involve seeking permission to address the topic [32]. Providing clear and accurate informational material is important, as some MWH reported searching for information themselves. Informational material could offer an easily accessible alternative to direct discussions. Providing information and raising awareness about the topic can also be done by a patient advocacy organization. Some men proposed using a questionnaire to screen for potential difficulties regarding sexual functioning and well-being in daily clinical care. Research also indicates that questionnaires can serve as tools to open up discussions about sensitive topics, such as sexual functioning and well-being [33]. Overall, normalizing the topic by reducing barriers, facilitating discussions, and providing informational material is crucial in clinical care, even if it may not be necessary to discuss sexual functioning at every outpatient visit.

A strength of this study is its qualitative nature, which enables the collection of in-depth insights into the lived experiences of MWH regarding sexual functioning and well-being that cannot be derived from quantitative data. These findings are needed in order to understand what aspects are important to measure, and to select an appropriate questionnaire. The involvement of a patient advocate strengthened the relevance of the study and provided valuable insight into its applicability. Additionally, the content of focus groups and semi-structured interviews were periodically evaluated by the interviewers to ensure the qualitative data was sufficient to meet the study´s aim. A limitation of the current study is the potential bias in the included MWH. A convenience sampling method was used, and the participating MWH may represent men who feel more comfortable discussing sexual functioning and well-being than other men. Furthermore, the study included a relatively small population, primarily consisting of Dutch MWH, as well as older MWH. The predominance of older participating MWH may reflect the assumption that sexual functioning and well-being is more commonly affected in elderly people, leading to greater interest in contributing to awareness. In contrast, younger MWH might be less likely to participate due to discomfort discussing their diagnosis or sexual functioning and well-being. Furthermore, sexual preference was not used as an inclusion or exclusion criterion, nor was it a specific focus of the focus groups and semi-structured interviews. However, it appeared that most participating men had a heteronormative preference, which could introduce bias.

A recommendation for future research is to explore lived experiences of younger MWH to better capture the perspectives of the broader patient population. Younger MWH might feel more comfortable sharing their experiences on an online forum. Next, considering different sexual orientations (e.g., gay) and different cultural or religious influences is recommended. Another area for consideration is the impact of HIV and HCV. While this study primarily reported the impact of HIV, future research should examine the effects of both infections, as they and their treatment may influence sexual health and well-being. Future research could also focus on developing tools or trainings for HCPs to address the topic of sexual functioning and well-being with MWH. Informational material and a questionnaire, such as the patient reported outcome measure (PROM) might be helpful. PROMs are questionnaires that assess aspects of health, reported directly from the patients’ perspective [34]. An example of such a generic PROM is the Patient-Reported Outcomes Measurement Information System (PROMIS^®^) Sexual Function and Satisfaction, which has a broader focus than only the binary assessment of being restricted in sexual functioning and measures multiple components of sexual functioning and well-being [35]. This generic PROM should be validated within the hemophilia population and can be supplemented with disease-specific questions based on the results of the current study. Considering the impact of hemophilia on sexual functioning and well-being, we suggest that drug developers consider measuring sexual functioning and well-being in both clinical trial and real-world settings.

## Conclusion

Concluding, this study highlights the need for greater awareness for the negative impact of hemophilia on sexual functioning and well-being, yet this impact is rarely discussed with HCPs. To address this gap, tools designed to provide and assess information about sexual functioning and well-being to normalize the topic and to open up discussions about it, particularly during specific life-phases, are necessary.

## Data Availability

The data that support the findings of this study are available from the corresponding author upon reasonable request.

## Abbreviations

MWH: Men with hemophilia
HIV: Human immunodeficiency virus
HCV: Hepatitis C virus
HRQOL: Health-related quality of life
HCP: Healthcare professional
HTC: Hemophilia treatment center
NVHP: Dutch hemophilia patient association (Nederlandse Vereniging voor Hemofilie Patiënten)
SPSS: Statistical package for social sciences
PROM: Patient-reported outcome measure
PROMIS: Patient-reported outcomes measurement system
